# Identification of Factors Influencing Donor-Derived Cell-Free DNA Levels up to One Year After Kidney Transplant

**DOI:** 10.1155/joot/7673476

**Published:** 2024-12-31

**Authors:** Ahmad Mirza, Imran Gani, Imran Parvez, Cari Weaver, Laura Mulloy, Rajan Kapoor

**Affiliations:** Medical College of Georgia, Augusta University Hospital and Medical Center, 1120 15th Street, Augusta AD 3401, Georgia

## Abstract

**Introduction:** Donor-derived cell-free DNA (dd-cfDNA) in the peripheral blood of allograft recipients has shown to early identify allograft injury. In this study, we assessed the factors that influence the amount of circulating dd-cfDNA during the first month postkidney transplant as well as its longitudinal trend.

**Materials and Methods:** A consecutive series of 98 adult kidney transplant recipients at a single center between July 2018 and January 2020 were included in this study. All demographic and operative details were collected for donors and recipients of the organ transplant. Median eGFR, dd-cfDNA, and serum creatinine were calculated at 1, 2, 3, 6, and 12 month posttransplant. Descriptive statistics were used for patient demographics. Nonparametric comparisons of dd-cfDNA cumulative distributions between dichotomized groupings were evaluated using Kruskal–Wallis or Mann–Whitney *U* tests.

**Results:** The median age of recipients was 54.5 years (IQR: 42.7–62.2). The cause of ESRD among recipients was hypertension (43%) and Type II diabetes mellitus (29%). Eighty-two percentage of patients received a deceased donor allograft, 14% received a living unrelated allograft, and 4% received a living related allograft. Sixteen percentage of recipients experienced delayed graft function (DGF). Median creatinine level at 1 month posttransplant was 1.75 mg/dL (IQR: 1.34–2.26) and median eGFR at 1 month posttransplant was 49.6 mL/min/1.73 m^2^ (IQR: 35–65). The median dd-cfDNA score at 1 month posttransplant for all recipients was 0.4% (IQR: 0.15–5.3). Donor sex was a statistically significant differential for dd-cfDNA score. Recipients from male donors had a significantly higher median dd-cf DNA score at 1 month posttransplant versus those who received a female kidney (0.57% vs. 0.28%, *p* < 0.01). Highest median score was recorded at the first month posttransplant (0.4%, IQR: 0.26–0.74), and a sustained downward trend was observed through Month 2 (0.19%, IQR: 0.17–0.31) and Month 3 (0.19%, IQR: 0.15–0.26). Correlation between 1-, 2-, 3-, 6-, and 12-month posttransplant median dd-cfDNA scores between deceased donor and living donor (LRD and LURD) cohorts was not statistically significant.

**Conclusion:** This study provides further insight into donor and recipient variables' effects on dd-cfDNA in the early posttransplant phase by analyzing a more diverse cohort of patients and adds to the knowledge around interpreting dd-cfDNA scores with clinical correlation for posttransplant management.

## 1. Introduction

Regular monitoring of allograft health to capture subclinical allograft injury is key to the long-term survival of both the allograft and kidney transplant recipients. However, the current standard methods of allograft monitoring such as protocol biopsy, level of serum creatinine, formation of donor-specific antibodies (DSAs), proteinuria, and eGFR decline suffer from invasiveness, inaccuracy, nonspecificity, and/or lack of sensitivity [1, 2]. Currently, several noninvasive biomarker assays are commercially available, with varying degrees of validation and utility attributed in the literature.

One of the more well-known noninvasive biomarkers is circulating donor-derived cell-free DNA (dd-cfDNA) in the peripheral blood of allograft recipients which has shown to quantitatively assay for the degree of allograft injury [3–5]. Increases in the level of circulating dd-cfDNA correlate with severity of tissue injury; thus, dd-cfDNA serves as a biomarker for allograft injury and rejection [6–8]. The dd-cfDNA assay AlloSure (CareDx, Inc.; Brisbane, CA) was clinically validated in the 14-center prospective DART study (NCT02424227) for its utility in discriminating active rejection from no rejection [9]. More recently, the 7-center ADMIRAL study (NCT04566055) demonstrated that persistently elevated AlloSure score (defined as values ≥ 0.5%) can predict a > 25% decline in eGFR over 3 years [10]. In addition, dd-cfDNA (AlloSure) ≥ 0.5% is associated with a nearly 3-fold increase in the risk of *de novo* DSA development—with the rise in score observed at a median of 91 days prior to the detection of *dn*DSA [10]. Elevated dd-cfDNA has shown to correlate with both alloimmune and nonalloimmune causes of injury [10].

Multiple factors should be considered when interpreting circulating dd-cfDNA levels. One of the factors previously examined is donor/recipient demography [11, 12]. Demographic characteristics of the donor and recipient, such as race, age, sex, type of transplant (deceased vs. living donor), and *de novo* versus retransplantation procedures, have all been shown to have at least some influence on transplant outcomes [13]. However, the inclusion of demographic characteristics such as race, age, and gender in creatinine-based eGFR calculation has been well documented [14–19]. Several studies have shown worse outcomes for both patient and allograft survival for African-American deceased donor kidney recipients [20–22].

In this study, we assess the factors that influence the amount of circulating dd-cfDNA during the first month posttransplant as well as the longitudinal trend of dd-cfDNA in patients that underwent renal transplant at Augusta Medical Center from July 2018 to January 2020.

## 2. Materials and Methods

This is a prospective observational study. Data collected included recipient and donor demographic details, cause of ESRD, transplant type, delayed graft function (DGF) status, and dd-cfDNA fraction (%). This study was approved by the Institutional Review Board (1587930-12), Augusta University Medical Center, Georgia, USA. The study design, analysis of data, and writing of the manuscript were performed by the authors. The study was externally funded by a grant from CareDX, Inc (California, USA). However, the funding organization had no role in design, analysis of results, and authorship for this study. A consecutive series of kidney transplants performed between July 2018 and January 2020 were included in this study. The following exclusion criteria were applied:

1. Pregnant recipients
2. Dual organ recipients
3. Recipient aged less than 18 years.

Standard induction immunosuppression was thymoglobulin (4.5 mg/kg, divided into three doses). The first dose was administered with premedicines (acetaminophen and diphenhydramine). The low-risk patients, defined as age greater than 70 years, history of cancer, and very low antibody titers, were induced with anti-IL2 receptor blocker (basiliximab). Patients were initiated on maintenance immunosuppression immediately posttransplant which consisted of an oral steroid taper dose, tacrolimus, and mycophenolate mofetil. All patients were followed up for a minimum of one year posttransplant at AU Medical Center transplant facility. Patients visited posttransplant clinics at 14 days and 1, 2, 3, 6, 9, and 12 months. At each clinic visit, routine renal function tests and tacrolimus levels were monitored. Assessment of dd-cfDNA was performed at 14 days and 1, 2, 3, 6, 9, and 12 months after kidney transplant (CareDx, Brisbane, CA). All serum samples were tested for dd-cfDNA as per the manufacturer's recommendations.

### 2.1. Statistical Analyses

Descriptive statistics were used for patient demographics, and distribution of dd-cfDNA measurements was obtained from the blood samples at the time of clinical events. Nonparametric comparisons of dd-cfDNA cumulative distributions between dichotomized groupings were evaluated using Kruskal–Wallis or Mann–Whitney *U* tests. All probability (*p*) values were two-tailed, and *p* < 0.05 was considered statistically significant.

## 3. Results

A total of 98 kidney transplant patients who received transplant between July 2018 and January 2020 at Augusta Medical Center were included in this study. All adult single-organ recipients between these dates with an available dd-cfDNA result at 1 month were included in the study.

### 3.1. Demographic Characterization

Table 1 shows the demographic characteristics of donors and recipients. For recipient ethnicity, 30% were Caucasian, 68% were African-American, 1% were Hispanic, and 1% were another race. For recipient sex, 61% were male and 39% were female. The median age of recipients was 54.5 years (IQR: 42.7–62.2). The highest cause of ESRD among recipients was hypertension (43%) followed by Type II diabetes mellitus (29%). Eighty-two percentage (*n* = 80) of patients received a deceased donor allograft, 14% (*n* = 14) received a living unrelated allograft, and 4% (*n* = 4) received a living related allograft. Sixteen percentage of recipients experienced DGF. Median creatinine level at 1 month posttransplant was 1.75 mg/dL (IQR: 1.34–2.26), and median eGFR at 1 month posttransplant was 49.6 mL/min/1.73 m^2^ (IQR: 35–65). For donor ethnicity, 65% were Caucasian, 27% were African-American, 5% were Hispanic, and 3% were multiracial. The donor sex distribution was 61% male and 39% female patients. Fourteen percentage of deceased donors were donation after cardiac death (DCD) donors. The median age of the donor was 46.6 years (range: 6–72), and the median KDPI was 47% (range: 1–99). The median cold ischemic time and warm ischemic time were 16.9 h (range: 0.58–35.27) and 35 min (range: 13–99), respectively. The median dd-cfDNA score at 1 month posttransplant for all recipients was 0.4% (IQR: 0.26–0.73).

### 3.2. Effects of Donor/Recipient Variables on Median dd-cfDNA Score at One Month Posttransplant

**Figure 1:**
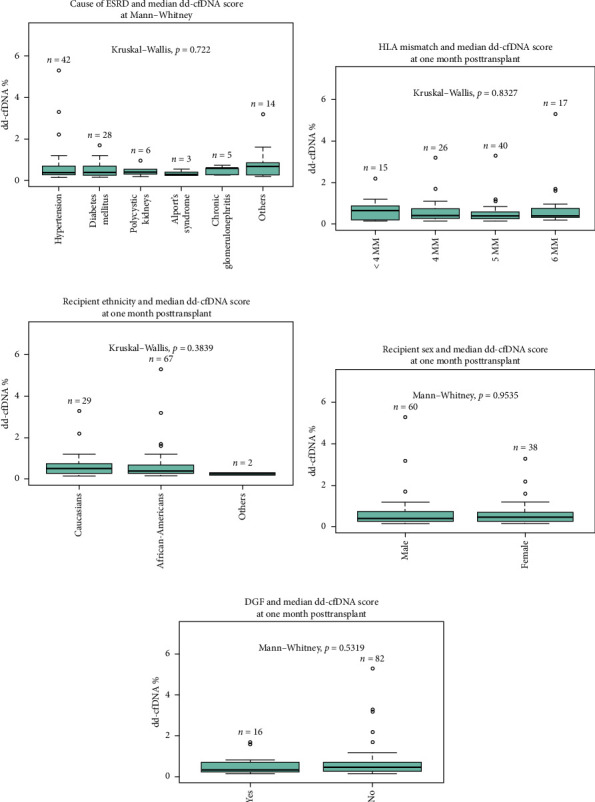
Effects of recipient demographics on median dd-cfDNA level 1 month posttransplant. (a) Cause of ESRD and median dd-cfDNA score at 1 month posttransplant. (b) HLA mismatch and median dd-cfDNA score at 1 month posttransplant. (c) Recipient ethnicity and median dd-cfDNA score at 1 month posttransplant. (d) Recipient sex and median dd-cfDNA score at 1 month posttransplant. (e) DGF status and median dd-cfDNA score at 1 month posttransplant.

Primary indication for transplant (cause of ESRD) did not significantly affect the recipient median dd-cfDNA score at 1 month posttransplant (hypertension 0.37%, diabetes mellitus 0.39%, polycystic kidney disease 0.40%, Alport's syndrome 0.28%, glomerulonephritis 0.59%, and other 0.67%, *p* = 0.072) (Figure 1(a)). The immunological characteristics assessed by the degree of HLA mismatch (MM) also did not significantly affect the median dd-cfDNA score at 1 month posttransplant (< 4MM 0.65%, 4MM 0.415%, 5MM 0.395%, 6MM 0.4%; *p* = 0.83) (Figure 1(b)). There was no statistically significant correlation between pretransplant calculated panel reactive antibodies (cPRA) and dd-cfDNA score at 1 month posttransplant.

There was no statistically significant correlation between different ethnicities (Caucasian 0.51%, African-American 0.39%, and others 0.25%, *p*=0.38) and sex (male 0.4% vs. female 0.46%, *p*=0.95) of recipients with dd-cfDNA score at 1 month posttransplant (Figures 1(c) and 1(d)). Sixteen percentage of all recipients experienced DGF; however, there was no statistical difference between DGF vs non-DGF recipients in terms of median dd-cfDNA score at 1 month posttransplant (0.32% vs. 0.46%, *p*=0.53) (Figure 1(e)).

**Figure 2:**
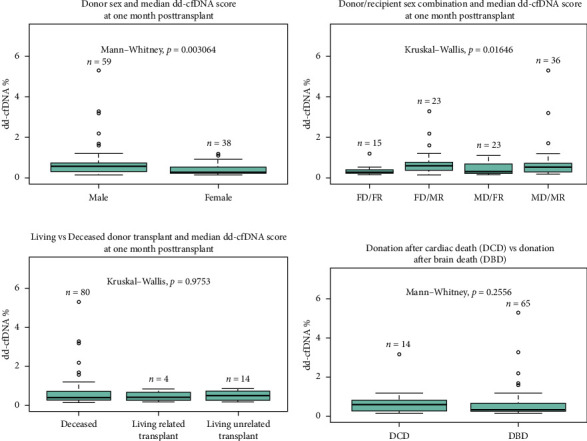
Effects of donor demographics on median dd-cfDNA level 1 month posttransplant. (a) Donor sex and median dd-cfDNA score at 1 month posttransplant. (b) Donor/recipient combination and median dd-cfDNA score at 1 month posttransplant. FD/FR = female donor to female recipient, FD/MR = female donor to male recipient, MD/FR = male donor to female recipient, MD/MR = male donor to male recipient. (c) Living vs. deceased donor transplant. LRD = living related donor, LURD = living unrelated donor. (d) Donation after cardiac death (DCD) vs. donation after brain death (DBD).

Sex of the donor was a significant differential for dd-cfDNA score. In this cohort, patients who received a kidney from a male donor had significantly higher median dd-cfDNA scores at 1 month posttransplant compared to those who received a kidney from a female donor (0.57% vs. 0.28%, *p* < 0.01) (Figure 2(a)). Further breakdown of donor–recipient sex combinations demonstrated higher median dd-cfDNA scores for male recipients than for female recipients (FD/FR 0.28%, FD/MR 0.59%, MD/FR 0.31%, and MD/MR 0.52%, *p*=0.016) (Figure 2(b)). On the contrary, donation type (deceased donor, living related donor, and living unrelated donor) did not significantly differ in median dd-cfDNA score at 1 month posttransplant (0.4% vs. 0.425% vs. 0.505%, *p*=0.975) (Figure 2(c)). Similarly, comparison of DCD and donation after brain death (DBD) in the deceased donor cohort did not yield statistical differences in median dd-cfDNA score in the first month posttransplant (0.61% vs. 0.34%, *p*=0.256 (Figure 2(d)).

Both cold and warm ischemia times of deceased donor kidney did not show statistically significant differences in median dd-cfDNA score at 1 month posttransplant. Deceased donor kidney KDPI also did not significantly influence median dd-cfDNA score in the first month posttransplant.

### 3.3. Longitudinal Analysis of dd-cfDNA and Clinical Indicators in the Acute Posttransplant Phase

**Figure 3:**
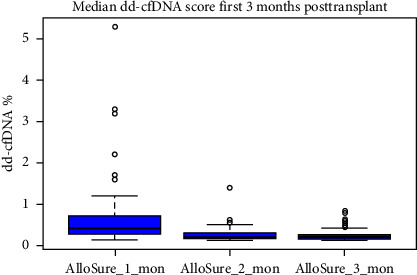
Median dd-cfDNA score first 3 months posttransplant.

**Figure 4:**
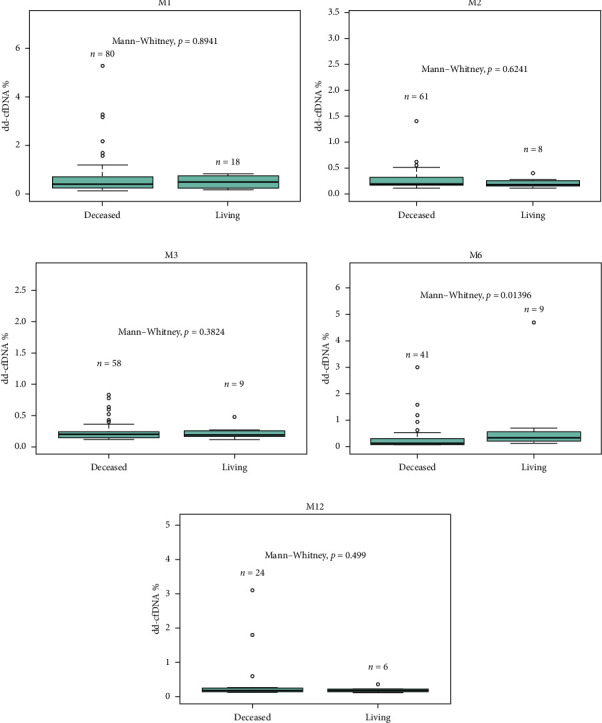
Longitudinal dd-cfDNA for Month 1, 2, 3, 6, and 12 posttransplant with transplant type.

Median dd-cfDNA score in the first 3 months posttransplant was similar to the previously published data [7] (Figure 3). Median score was the highest at the first month posttransplant (0.4%, IQR: 0.26–0.74), and a sustained downward trend was observed through Month 2 (0.19%, IQR: 0.17–0.31) and Month 3 (0.19%, IQR: 0.15–0.26). The correlation between 1-, 2-, 3-, 6-, and 12-month posttransplant median dd-cfDNA scores were compared between deceased donor and living donor (LRD and LURD) cohorts. There was a statistically significant difference seen only at Month 6 (deceased 0.15% vs. living 0.31%) (Figure 4).

Median eGFR and serum creatinine were calculated for 1, 2, 3, 6, and 12 month posttransplant in three dd-cfDNA score cohorts: ≤ 0.5%, 0.51%–0.99%, and ≥ 1.0% for all transplant recipients where the paired dd-cfDNA scores were available. There was no statistically significant difference between the three groups. Furthermore, analysis of all patients with recorded BK polyomavirus PCR copy number in either the urine or serum at any point within 12 month posttransplant showed no correlation between AlloSure score and BK PCR copy number.

### 3.4. Rejection and Injury Episodes Within First Year Posttransplant

For-cause biopsies were performed for 8 patients with paired dd-cfDNA scores during the first year posttransplant. Of those 8 patients, 2 patients had rejection episodes (Table 2). One patient with acute T-cell–mediated rejection (Banff IIA) and active antibody-mediated rejection had an elevated dd-cfDNA score and serum creatinine at the time of biopsy (3.8%, 3.8 mg/dL). One patient with borderline acute cellular rejection with absence of antibody-mediated rejection did not have elevated dd-cfDNA at the time of biopsy but did present with high serum creatinine (0.34%, 4.72 mg/dL). Of 5 patients who underwent biopsies but did not have biopsy-proven rejection, 4 patients had low dd-cfDNA score (0.12%–0.22%). One patient with dd-cfDNA score above 0.5% was diagnosed with acute tubular injury (ATI) (0.62%). The median time of graft survival was 372.9 days (IQR: 42.49–645.76).

## 4. Discussion

The early detection of allograft injury is critical for the long-term survival of both the allograft and recipient. Chong et al. contend that it is the underlying risk factors associated with race/ethnicity (such as genetic variation) that primarily affect outcomes, rather than the donor race itself [23]. Although the incidence of acute rejection within 1 year posttransplantation is less frequent in kidney transplants compared to other organs, the current methods to detect allograft rejection lack sensitivity to detect early allograft injury [24]. Noninvasive biomarkers such as dd-cfDNA could allow clinicians to detect subclinical rejection, which can currently only be detected with invasive protocol biopsy. The invasive protocol biopsy carries an elevated risk of iatrogenic harm to both the graft and the recipient and is subject to reader interpretation heterogeneity [6, 25, 26]. Several publications have evaluated the role of dd-cfDNA to monitor allograft injury and rejection [3, 7–10]. However, the relationship between donor/recipient variables that affect the level of dd-cfDNA in kidney transplant patients has only recently been investigated. Anand et al. demonstrated associations between repeat transplant, dual transplant, DCD donor, and PRA with higher dd-cfDNA at 1 month posttransplant [11]. Also, a significant downtrend of longitudinally obtained dd-cfDNA scores was observed during the first 3 months posttransplant [11]. Furthermore, Lane, Nie, and Kayler demonstrated early elevation of dd-cfDNA scores was not necessarily prognostic of long-term graft or patient survival outcomes in a single-center cohort of surveilled patients but may presage elevated risk of acute rejection [27]. In this study, we further investigated the association between demographic characteristics of both donors and recipients on dd-cfDNA score at the first month posttransplant, as well as the longitudinal course of median dd-cfDNA score.

We have analyzed 98 kidney transplant patients for the association between donor and recipient ethnicity, sex, age, cause of ESRD, transplant type, DGF, and dd-cfDNA score at 1 month posttransplant. In addition, we assessed the longitudinal change in dd-cfDNA score based on measurements obtained up to 12 months posttransplant. Our study population differs from the population by Anand et al. with a significantly higher percentage of African-American patients (African-American 68% and Caucasian 30%), while Anand et al.'s study population consisted of mostly Caucasian patients (African-American 2% and Caucasian 89.6%) [11]. This represents a significant shift in the study demography and a potential for differential study outcomes given the prior work around donor race/ethnicity-based differences in deceased donor outcomes [20–22].

Several studies have reported median dd-cfDNA values at 1 month posttransplant for stable patients. Anand et al. reported a median dd-cfDNA value of 0.32% (IQR: 0.26%–0.55%) [11], Bromberg et al. reported a median dd-cfDNA value of 0.21% (IQR: 0.12%–0.39%) [28], and Sigdel et al. reported a median dd-cfDNA value of 0.4% (IQR: 0.03%–6.8%) [29]. In our cohort, the median value of dd-cfDNA at 1 month posttransplant was 0.4% (IQR: 0.26%–0.74%) (Figure 3). These values are all nominally similar and suggest reproducibility across cohorts with broad differences in demography as well as center-specific transplant management.

No association with dd-cfDNA value was observed between recipients' age, sex, ethnicity, DGF, HLA MM, cold ischemic time, warm ischemic time, and transplant type.

In our study cohort, patients who received a male donor kidney had a statistically higher dd-cfDNA median score in the first month posttransplant compared to those who received a female donor kidney (0.57% vs. 0.28%, *p* < 0.001). The number of deceased male donations was 55.1% (54/98), and the number of deceased female donations was 25.5% (25/98). The number of living female donors (both LRD and LURD) was 12.2% (12/98), while the number of living male donors (both LRD and LURD) was 7.1% (7/98). Whether the higher median dd-cfDNA at 1 month posttransplant for the recipients of male donor kidney is partially due to the higher percentage of male deceased donors requires further examination in a larger study cohort. Recent data reported by Congress Craig, Tennankore, and Vinson demonstrated that female recipients of male donor kidneys are at increased risk for graft failure compared with other donor/recipient sex pair due to HY minor histocompatibility that female recipients are not exposed [30]. The study also showed that prior transplant with a male donor results in anti-HY antibody formation that impacts subsequent transplant outcomes and that the prior or current donor sex pairing was significantly associated with death-censored graft loss when stratified by recipient age at the time of retransplant [30].

Statistically significant differences in median dd-cfDNA score at 1 month posttransplant in deceased versus living donor transplants have been reported in a previous study by Anand et al. [11]. The study demonstrated an association with higher dd-cfDNA with DCD donors compared to LRD or LURD, and this difference was attributed to ischemia–reperfusion injury in DCD kidneys immediately after transplant [4, 11]. Contrary to the previous findings, our study cohort showed no statistically significant difference between deceased, LRD, and LURD in terms of the first month median dd-cfDNA score (0.4% vs. 0.425% vs. 0.505%, *p*=0.975). A numerically higher dd-cfDNA score was observed in LURD and LRD compared to deceased donors (Figure 2(c)). This finding could be due to the small sample size of LRD (*n* = 4) and LURD (*n* = 14) recipients compared to deceased donor recipients (*n* = 80). Also, patients who received DBD vs DCD organ did not show a statistically significant difference in dd-cfDNA score (Figure 2(d)). A further analysis using more numerically balanced cohorts of deceased, LRD, and LURD recipients needs to be performed to confirm the findings of this study.

A previous study showed a statistically significant difference and a positive correlation between cPRA and the level of dd-cfDNA [11]. Our study showed no statistical significance between pretransplant cPRA and dd-cfDNA value. The correlation between preexisting DSA and cPRA with dd-cfDNA level has not been studied extensively, and further studies focusing on this correlation would provide valuable information on the clinical interpretation of dd-cfDNA.

dd-cfDNA level at the first month tends to be higher due to ischemia–reperfusion injury or residual surgical trauma, and the dd-cfDNA level usually returns to a baseline by 3 months posttransplant for stable patients. Median dd-cfDNA score for the first 3 months posttransplant was examined in this study cohort, and a downward trend from the first month posttransplant to 3 month posttransplant was observed (Figure 3), in concordance with reports from previous studies [11, 27, 31]. The patient's elevated dd-cfDNA score was not correlated with BK nephropathy (defined as the viral load in excess of 10,000 copies/mL). Further studies using a larger number of samples need to be performed to confirm this finding. Similarly, no statistically significant correlation was observed between serum creatinine, eGFR, and median dd-cfDNA values for 1, 2, 3, 6, and 12 month posttransplant.

The correlation between rejection and injury with dd-cfDNA score showed that the patient with acute T-cell–mediated rejection (Banff IIA) and active antibody-mediated rejection had an elevated dd-cfDNA score and serum creatinine at the time of biopsy (3.8%, 3.8 mg/dL). The patient with borderline cellular rejection without evidence of antibody-mediated rejection and the patient with ATI did not show elevated dd-cfDNA scores. The case of ATI was specifically noted to be in the absence of allograft rejection, suggesting dd-cfDNA release might be limited.

The primary limitation of this study is a single-center study with retrospective observation and a limited number of cohorts of patients.

In conclusion, this study provides further insight into donor and recipient variables' effects on dd-cfDNA by analyzing a more diverse cohort of patients. Although further studies using a larger sample size for all analysis groups need to be conducted to evaluate the findings of this study, the impact of donor variables on dd-cfDNA in the early posttransplant phase adds to the knowledge around interpreting dd-cfDNA scores with clinical correlation for posttransplant management.

## Figures and Tables

**Table 1:** Demographics of donors and recipients.

Recipients demographics	Category	Percentage
Ethnicity	Caucasians	30% (29)
	African-Americans	68% (67)
	Hispanics	1% (1)
	Others	1% (1)

Sex	Male	61% (60)
	Female	39% (38)

Cause of ESRD	Hypertension	43% (42)
	Diabetes mellitus—Type II	29% (28)
	Polycystic kidneys	6% (6)
	Alport's syndrome	3% (3)
	Glomerulonephritis	5% (5)
	IgA nephropathy	2% (2)
	Systemic lupus erythematosus	1% (1)
	Congenital posterior urethral valves	2% (2)
	Wegener's granulomatosis	0% (0)
	Bartter's sydrome	1% (1)
	Focal segmental glomerulosclerosis (FSGS)	3% (3)
	Membranous nephropathy	2% (2)
	Others	3% (3)

Organ	Right kidney	55% (54)
	Left kidney	45% (44)

Transplant type	Living related	4% (4)
	Living unrelated	14% (14)
	Deceased	82% (80)

DGF	Yes	16% (16)
	No	84% (82)

Rejection	Active ABMR Banff Grade 2A	1% (1)
	Acute TCMR Banff Grade 1B	1% (1)
	Acute TCMR Banff Grade 2A	1% (1)
	Borderline TCMR	1% (1)

Age	54.5 (IQR: 42.7–62.2)	
Creatinine Month 1	1.75 mg/dL (IQR: 1.3–2.3)	
eGFR Month 1	49.6 mL/min/1.73 m^2^ (IQR: 35–65)	
Graft survival day	372.5 (IQR: 239.9–524.8)	
dd-cfDNA Month 1	Median 0.4 (IQR: 0.26–0.73)	

**Donor demographics**	**Category**	**Percentage**

Ethnicity	Caucasians	65% (64)
	African-Americans	27% (26)
	Hispanics	5% (5)
	Multiracial	3% (3)

Sex	Male	61% (59)
	Female	39% (38)

DCD status	No	67% (65)
	Yes	14% (14)
	Unknown	19% (19)

Cause of death	Head trauma	26% (25)
	Anoxia	24% (24)
	Stroke (CVA)	28% (27)
	Other	4% (4)
	Not specified	18% (18)

HLA mismatch	0	2% (2)
	1	1% (1)
	2	5% (5)
	3	7% (7)
	4	27% (26)
	5	41% (40)
	6	17% (17)

Age	46.6 (IQR: 28.2–57.2)	
Warm ischemic time (min)	35 (13–99)	
Cold ischemic time (hr)	16.9 (: 0.58–35.27)	
Terminal creatinine	0.9 mg/dL (0.37–7.5)	
KDPI	47% (1–99)	

**Table 2:** Rejection and Injury episodes within first year posttransplant.

Rejection				
Transplant date	Biopsy date	AlloSure score at biopsy (%)	Serum creatinine at biopsy (mg/dL)	Pathology
12/29/2019	1/16/2020	3.20	3.8	Acute T-cell–mediated rejection, Banff IIA, active antibody–mediated rejection
1/30/2020	3/26/2020	0.34	4.72	Borderline for acute cellular rejection, negative for antibody mediated rejection

**Injury**				
**Transplant date**	**Biopsy date**	**AlloSure score at biopsy (%)**	**Pathology**	

12/20/2018	1/18/2019	0.12	Mild tubular epithelial injury	
12/25/2018	1/7/2019	0.14	Tubular epithelial injury. Mild arterial sclerosis, donor disease	
3/16/2019	3/29/2019	0.22	Thrombotic microangiopathy in association with geographic cortical necrosis	
4/17/2019	7/23/2019	0.17	Inflamed and cystic dilation of renal pelvis and calyces associated with chronic interstitial inflammation and fibrosis	
6/6/2019	7/29/2019	0.62	Acute tubular injury	

## Data Availability

All data are saved at Augusta University Central Archives. All data can be made available in an anonymized manner on request.

## References

[1] Nickerson P. (2009). Post-Transplant Monitoring of Renal Allografts: Are We There yet?. *Current Opinion in Immunology*.

[2] Josephson M. A. (2011). Monitoring and Managing Graft Health in the Kidney Transplant Recipient. *Clinical Journal of the American Society of Nephrology*.

[3] Thongprayoon C., Vaitla P., Craici I. M. (2020). The Use of Donor-Derived Cell-Free DNA for Assessment of Allograft Rejection and Injury Status. *Journal of Clinical Medicine*.

[4] Oellerich M., Sherwood K., Keown P. (2021). Liquid Biopsies: Donor- Derived Cell- Free DNA for the Detection of Kidney Allograft Injury. *Nature Reviews Nephrology*.

[5] Knight S. R., Thorne A., Lo Faro M. L. (2019). Donor-Specific Cell-Free DNA as a Biomarker in Solid Organ Transplantation. A Systematic Review. *Transplantation*.

[6] Gielis E. M., Ledeganck K. J., De Winter B. Y. (2015). Cell-Free DNA: An Upcoming Biomarker in Transplantation. *American Journal of Transplantation*.

[7] Snyder T. M., Khush K. K., Valantine H. A., Quake S. R. (2011). Universal Noninvasive Detection of Solid Organ Transplant Rejection. *Proceedings of the National Academy of Sciences of the United States of America*.

[8] De Vlaminck I., Valantine H. A., Snyder T. M. (2014). Circulating Cell-Free DNA Enables Noninvasive Diagnosis of Heart Transplant Rejection. *Science Translational Medicine*.

[9] Bloom R. D., Bromberg J. S., Poggio E. D. (2017). Cell-Free DNA and Active Rejection in Kidney Allografts. *Journal of the American Society of Nephrology*.

[10] Bu L., Gupta G., Pai A. (2022). Clinical Outcomes From the Assessing Donor-Derived Cell-Free DNA Monitoring Insights of Kidney Allografts With Longitudinal Surveillance (ADMIRAL) Study. *Kidney International*.

[11] Anand S., Lopez-Verdugo F., Sanchez-Garcia J. (2021). Longitudinal Variance of Donor-Derived Cell-Free DNA (Dd-cfDNA) in Stable Kidney Transplant (KTx) Patients Are Influenced by Donor/Recipient Variables. *Clinical Transplantation*.

[12] Urban M., Booth K., Jungschleger J., Netuka I., Schueler S., MacGowan G. (2019). Impact of Donor Variables on Heart Transplantation Outcomes in Mechanically Bridged Versus Standard Recipients. *Interactive Cardiovascular and Thoracic Surgery*.

[13] Hariharan S., Israni A. K., Danovitch G. (2021). Long-Term Survival After Kidney Transplantation. *New England Journal of Medicine*.

[14] Fontanarosa P. B., Bauchner H. (2018). Race, Ancestry, and Medical Research. *JAMA*.

[15] Eneanya N. D., Yang W., Reese P. P. (2019). Reconsidering the Consequences of Using Race to Estimate Kidney Function. *JAMA*.

[16] Delgado C., Baweja M., Burrows N. R. (2021). Reassessing the Inclusion of Race in Diagnosing Kidney Diseases: An Interim Report From the NKF-ASN Task Force. *Journal of the American Society of Nephrology*.

[17] Hsu J., Johansen K. L., Hsu C. Y., Kaysen G. A., Chertow G. M. (2008). Higher Serum Creatinine Concentrations in Black Patients With Chronic Kidney Disease: Beyond Nutritional Status and Body Composition. *Clinical Journal of the American Society of Nephrology*.

[18] Swanson S. J., Hypolite I. O., Agodoa L. Y. C. (2002). Effect of Donor Factors on Early Graft Survival in Adult Cadaveric Renal Transplantation. *American Journal of Transplantation*.

[19] Callender C. O., Cherikh W. S., Miles P. V. (2008). Blacks as Donors for Transplantation: Suboptimal Outcomes Overcome by Transplantation Into Other Minorities. *Transplantation Proceedings*.

[20] Molnar M. Z., Kovesdy C. P., Bunnapradist S. (2013). Donor Race and Outcomes in Kidney Transplant Recipients. *Clinical Transplantation*.

[21] Callender C. O., Cherikh W. S., Traverso P., Hernandez A., Oyetunji T., Chang D. (2009). Effect of Donor Ethnicity on Kidney Survival in Different Recipient Pairs: An Analysis of the OPTN/UNOS Database. *Transplantation Proceedings*.

[22] Locke J. E., Warren D. S., Dominici F. (2008). Donor Ethnicity Influences Outcomes Following Deceased-Donor Kidney Transplantation in Black Recipients. *Journal of the American Society of Nephrology*.

[23] Chong K., Litvinovich I., Chen S. S., Zhu Y., Argyropoulos C., Ng Y.-H. (2021). Reconsidering Donor Race in Predicting Allograft and Patient Survival Among Kidney Transplant Recipients. *KIDNEY360*.

[24] Hart A., Lentine K. L., Smith J. M. (2021). OPTN/SRTR 2019 Annual Data Report: Kidney. *American Journal of Transplantation*.

[25] Nankivell B. J., Chapman J. R. (2006). The Significance of Subclinical Rejection and the Value of Protocol Biopsies. *American Journal of Transplantation*.

[26] Orandi B. J., Chow E. H., Hsu A. (2015). Quantifying Renal Allograft Loss Following Early Antibody-Mediated Rejection. *American Journal of Transplantation*.

[27] Lane R., Nie J., Kayler L. (2022). Donor-derived Cell-Free DNA as a Graft Injury Marker Following Kidney Transplantation. *Transplantation Direct*.

[28] Bromberg J. S., Brennan D. C., Poggio E. (2017). Biological Variation of Donor-Derived Cell-Free DNA in Renal Transplant Recipients: Clinical Implications. *The Journal of Applied Laboratory Medicine*.

[29] Sigdel T. K., Archila F. A., Constantin T. (2018). Optimizing Detection of Kidney Transplant Injury by Assessment of Donor-Derived Cell-Free DNA via Massively Multiplex PCR. *Journal of Clinical Medicine*.

[30] Craig S., Tennankore K., Vinson A. The Role of Donor Sex in Females Undergoing Repeat Kidney Transplant: Does Prior Donor Sex Matter?. *AJT*.

[31] Mirza A. Effect of Donor and Recipient Characteristics on Donor Derived Cell Free Dna Levels Upto One Year Post Kidney Transplant. https://atc.digitellinc.com/p/s/effect-of-donor-and-recipient-characteristics-on-donor-derived-cell-free-dna-levels-upto-one-year-post-kidney-transplant-34029.

